# Effects of growth trajectory of shock index within 24 h on the prognosis of patients with sepsis

**DOI:** 10.3389/fmed.2022.898424

**Published:** 2022-08-22

**Authors:** Fengshuo Xu, Luming Zhang, Tao Huang, Didi Han, Rui Yang, Shuai Zheng, Aozi Feng, Liying Huang, Haiyan Yin, Jun Lyu

**Affiliations:** ^1^Department of Intensive Care Unit, The First Affiliated Hospital of Jinan University, Guangzhou, China; ^2^Department of Nosocomial Infection Management, Luoyang Orthopedic-Traumatological Hospital, Orthopedics Hospital of Henan Province, Zhengzhou, China; ^3^School of Public Health, Xi’an Jiaotong University Health Science Center, Xi’an, China; ^4^Department of Clinical Research, The First Affiliated Hospital of Jinan University, Guangzhou, China; ^5^School of Public Health, Shaanxi University of Chinese Medicine, Xianyang, China; ^6^Guangdong Provincial Key Laboratory of Traditional Chinese Medicine Informatization, Guangzhou, China

**Keywords:** sepsis, shock index, growth trajectory, prognosis, latent growth mixture modeling

## Abstract

**Background:**

Sepsis is a serious disease with high clinical morbidity and mortality. Despite the tremendous advances in medicine and nursing, treatment of sepsis remains a huge challenge. Our purpose was to explore the effects of shock index (SI) trajectory changes on the prognosis of patients within 24 h after the diagnosis of sepsis.

**Methods:**

This study was based on Medical Information Mart for Intensive Care IV (MIMIC- IV). The effects of SI on the prognosis of patients with sepsis were investigated using C-index and restricted cubic spline (RCS). The trajectory of SI in 24 h after sepsis diagnosis was classified by latent growth mixture modeling (LGMM). Cox proportional hazard model, double robust analysis, and subgroup analysis were conducted to investigate the influence of SI trajectory on in-hospital death and secondary outcomes.

**Results:**

A total of 19,869 patients were eventually enrolled in this study. C-index showed that SI had a prognostic value independent of Sequential Organ Failure Assessment for patients with sepsis. Moreover, the results of RCS showed that SI was a prognostic risk factor. LGMM divided SI trajectory into seven classes, and patients with sepsis in different classes had notable differences in prognosis. Compared with the SI continuously at a low level of 0.6, the SI continued to be at a level higher than 1.0, and the patients in the class whose initial SI was at a high level of 1.2 and then declined had a worse prognosis. Furthermore, the trajectory of SI had a higher prognostic value than the initial SI.

**Conclusion:**

Both initial SI and trajectory of SI were found to be independent factors that affect the prognosis of patients with sepsis. Therefore, in clinical treatment, we should closely monitor the basic vital signs of patients and arrive at appropriate clinical decisions on basis of their change trajectory.

## Background

Sepsis is a serious disease with high morbidity and mortality in clinic. It is caused by pathogenic infection wherein host immune regulation is out of control, resulting in dysregulated immune response and strong inflammatory reaction, both of which can lead to tissue damage, organ dysfunction and even failure, and finally lead to death (1). A study that investigated the global burden of sepsis estimated that there are 48.9 million sepsis cases and 11 million sepsis-related deaths worldwide each year (2). Owing to their complicated condition and unstable vital signs, patients with sepsis often must be admitted to the intensive care unit (ICU) for close monitoring and treatment. A meta-analysis found that the mortality rate of patients with sepsis treated in ICUs is approximately 40% (3). A prospective study from Brazil estimated that the annual rate of adult cases of sepsis treated in ICUs is 290 per 100,000 people, resulting in about 420,000 cases each year, of which 230,000 die in the hospital (4). Despite the tremendous advances in medicine and nursing, the definition, identification, and proper treatment of sepsis remain a huge challenge. Therefore, early detection of infection in patients with sepsis, accurate judgment of disease severity, effective prediction of prognosis, and early intervention are the key to improving the cure rate and reducing the mortality rate.

Shock index (SI) is calculated from heart rate (min^–1^)/systolic blood pressure (mmHg), the use of which is non-invasive, simple, and convenient and allows repeated dynamic monitoring. A prospective cohort study revealed that compared with routine vital signs, SI is an independent factor predicting postpartum hemorrhage and postpartum sepsis (5). Another study examined 425,808 acute stroke cases and found that SI was an important predictor of outcomes of patients with acute stroke, including mortality and moving state when discharged (6). In addition, SI has been shown to be a reliable predictor of death in patients with sepsis in the emergency department (7, 8). Changes in the condition of sepsis are a dynamic process, and the relationship between changes in blood pressure and heart rate and changes in the patient’s condition and treatment is complicated. Previous studies on patients with sepsis were cross-sectional in nature, and studies on the longitudinal trajectory prediction of important indicators of patients are relatively few. Between these types of studies, the latter has greater clinical significance. In particular, predicting the prognosis of patients through changes in patient vital signs is more clinically meaningful.

The changes of short-term SI in patients with sepsis after diagnosis may reflect different prognostic status. In this study, we used the large public database Medical Information Mart for Intensive Care IV (MIMIC-IV) to explore the impact of SI trajectory changes on the prognosis of patients within 24 h after the diagnosis of sepsis.

## Methods

### Data source

The data used in this study were derived from the MIMIC-IV database, which is a large, openly accessible and relational database (9, 10). It was founded with funding from the National Institutes of Health by emergency physicians, critical care physicians, and computer science experts from Beth Israel Deaconess Medical Center, Massachusetts Institute of Technology, Oxford University, and Massachusetts General Hospital. MIMIC-IV contains comprehensive and high-quality data on critically ill patients admitted to Beth Israel Deaconess Medical Center in Boston, Massachusetts, United States, from 2008 to 2019 (11). The database is annually updated, with the latest version being MIMIC-IV 1.0 released in March 2021. After completing the necessary training, we were allowed access to the database (Record ID: 38455175).

### Inclusion and exclusion criteria

According to the Sepsis 3.0 diagnostic criteria, sepsis is defined as a suspected infection accompanied by an acute increase of ≥2 points in the Sequential Organ Failure Assessment (SOFA) score (12, 13). We used this definition to identify patients with sepsis. If sepsis occurs more than once during the same hospitalization, only the information for the first diagnosis was used.

The exclusion criteria were as follows: (a) patients aged < 18 years; (b) patients who died within 24 h of diagnosis of sepsis; (c) patients who did not meet the requirement that SI was measured at least once every 4 h within 24 h after diagnosis of sepsis; (d) patients who had the proportion of missing value of covariates > 20%; and (e) patients whose SI was measured at outliers three times the interquartile interval of the overall measurement.

After identifying the study population, we used Structured Query Language programming by Navicat Premium 11.2.7.0 to extract the required data based on their stay_id and hadm_id.

### Exposure factor

The exposure factor adopted in this study was the trajectory of SI within 24 h following the diagnosis of sepsis. SI was calculated using the equation SI = heart rate (min^–1^)/systolic pressure (mmHg). We could not guarantee that the heart rate and systolic pressure of the patients in the MIMIC-IV were measured every hour. Thus, to ensure sufficient sample size for this study, we analyzed within a time unit of 4 h. Previous and subsequent analyses found that as the SI increases, the patient’s prognosis becomes worse. Thus, we used the maximum SI per time unit as the value for this period (14).

### Outcome indicators

The primary outcome of the present study was the occurrence of in-hospital death. Secondary outcomes included in-ICU death, 28-day death, ventilation-free days in 28 days, and vasopressor-free days in 28 days. Vasopressors included norepinephrine, epinephrine, dopamine, and dobutamine. Follow up began with the diagnosis of sepsis and ended with death or discharge.

### Covariates

All factors that might confuse the relationship between exposure factor and outcome indicators, including demographics, disease severity scores, laboratory tests, and treatment measures, were treated as covariates and adjusted in subsequent analysis. To reduce information bias, we excluded variables with a missing ratio of over 20%. The covariables finally determined were age, gender, ethnicity (white, black, and others), weight, first_care_unit (medical intensive care unit/surgical intensive care unit, coronary care unit, and others), SOFA, Charlson_comorbidity_index, use of ventilator, vasopressor and continuous renal replacement therapy (CRRT) during the first 24 h of sepsis diagnosis, anion gap, sodium, potassium, chloride, bicarbonate, phosphate, glucose, creatinine, blood urea nitrogen, red blood cells, white blood cells (WBC), platelets, red blood cell distribution, hemoglobin, pCO2, pO2, and pH. For the indicators measured multiple times during hospitalization, we took the first measurements after the diagnosis of sepsis. We used the “mice” package of R software to deal with missing values of covariables through multiple imputations. Supplementary Figure 1 shows the specific missing ratio of covariables before imputation.

### Prognostic value of shock index

We constructed Cox proportional hazard models containing only SOFA and both SOFA and initial SI. We then calculated and compared the C-indexes of the two models through the “compareC” package of R software to determine the predictive value of SI for in-hospital death. We then explored the dose-response relationship between SI and in-hospital death by using restricted cubic spline (RCS). We used the likelihood ratio test to examine the overall statistical association and the potential non-linear relationship (15).

### Latent growth mixture modeling

We used the “lcmm” package of R software to analyze the trajectory of SI by establishing latent growth mixture modeling (LGMM). LGMM assumes that the population consists of several potential categories, each having the same trajectory, characterized by the average contour of the trajectory (16). A key factor in establishing the LGMM model is that the number of potential classes must be specified. To select the best number of potential categories, we built quadratic growth models containing one to eight classes. The optimal number of potential classes is determined by the following: First, the lower the Akaike information criterion, Bayesian information Criteria (BIC), and sample-adjusted BIC are, the better the fitting effect will be (17). Second, the higher the log-likelihood ratio and entropy are, the better the fitting effect will be, and the entropy is not less than 0.7. Third, the sample size of each class shall not be less than 1% of the total sample size. Fourth, the average posterior probability of each class is not less than 70%. Finally, the parsimony and clinical interpretability of the model must be considered.

For the baseline characteristics of each trajectory class, the continuous variables conforming to normal distribution were described by means and standard deviations, and differences among classes were compared by ANOVA. Non-conforming variables were described by median and interquartile range, and differences between classes were compared by Kruskal–Wallis test. Categorical variables were described by frequency and percentage, and differences between classes were compared using Chi-square test or Fisher’s exact test.

### Predictive value of shock index trajectory to primary outcome

Five Cox proportional hazard models with different numbers and types of covariates were constructed to explore the impact of the trajectory of SI on the risk of in-hospital death. In model 5, in which all covariates were adjusted, different reference classes were set to compare the risk of in-hospital death for each trajectory class.

Inverse probability of treatment weighting (IPTW) based on the propensity score was applied to the survival analysis to minimize confounding bias, similar to a randomized controlled trial, to estimate directly the effects of exposure factors on outcome (12, 18). Using the “twang” package of R software, we constructed the propensity scoring model of the above covariables for trajectory classes via the method of gradient boosting machine (GBM). We obtained the most balanced weight of the covariables by applying the optimal iteration number estimation to the GBM model according to the stop rule standard of “es. Mean.” GBM is a machine learning approach that uses an iterative process with multiple regression trees to capture complex relationships between intervention conditions and baseline covariables (18). We then used the estimated propensity score multiplied by the proportion of each class in the original cohort as a stable weight to construct the IPTW cohort, a virtual population whose distribution of covariates is independent of trajectory classes. In this cohort, Cox proportional hazard models without any covariables adjusted, with only covariables still unbalanced [standardized mean difference (SMD) > 0.1 or *P* < 0.05] adjusted, and with all covariables adjusted were developed to achieve a double robust analysis to verify the stability of the results.

We also assessed the predictive value of the SI trajectory classes compared with the initial SI value for the risk of in-hospital death by comparing the C-indexes.

Furthermore, we performed stratified analysis according to age (< 65, ≥ 65), gender (female or male), SOFA (<3, ≥3), Charlson_comorbidity_index (<6, ≥6) and usage of vasopressors (to distinguish sepsis and septic shock according to their use or non-use) to assess potential modified effects. Moreover, we evaluated potential interactions by adding a cross-product term of class and the stratification variables described above to the model.

### Predictive value of shock index trajectory to secondary outcomes

We constructed multivariate Cox proportional hazard models or linear regression models to explore the effects of SI trajectory on secondary outcomes. Covariables other than ventilator use were adjusted in the model for ventilation-free days in 28 days. Covariables other than vasopressor use were adjusted in the model for ventilation-free days in 28 days. All of these covariables were adjusted in two other secondary outcome models.

**FIGURE 1:**
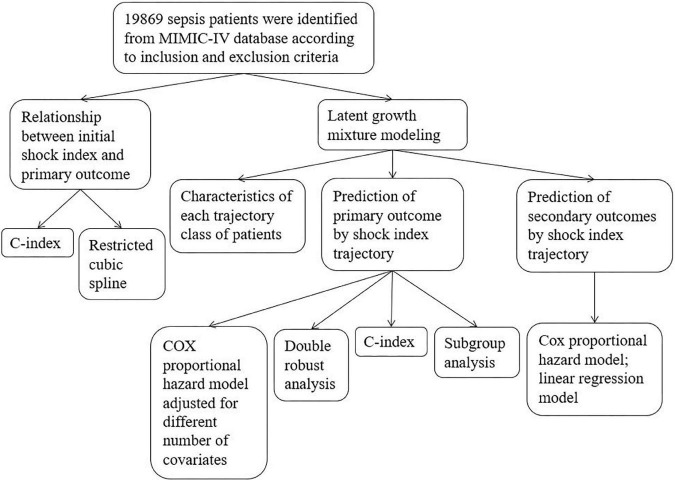
Flow chart. Primary outcome was in-hospital death; secondary outcomes included in-ICU death, 28-day death, ventilation-free days in 28 days, and vasopressor-free days in 28 days.

A two-sided *P* < 0.05 was considered statistically significant. All statistical analyses in this study were performed using R software (4.0.3). In order to more comprehensively and accurately analyze the impact of SI on the prognosis of patients with sepsis, this study adopted many statistical methods. For easy reading, we show the whole research process in Figure 1.

## Results

### Participants

A total of 19,869 patients were included in this study, and 3,058 (15.4%) of them died in the hospital. The median follow-up time was 7.83 (4.96, 14.04) days. In general, SI exhibited a downward trend. The specific baseline characteristics are given in Table 1.

**TABLE 1:** Baseline characteristics of seven classes.

Variable	Overall	Class 1	Class 2	Class 3	Class 4	Class 5	Class 6	Class 7	*P*-value
N	19,869	3,763	8,056	957	1,907	220	4,649	317	
Age, year	67.00 [56.00, 78.00]	70.00 [59.00, 80.00]	68.00 [58.00, 78.00]	67.00 [56.00, 77.00]	65.00 [53.00, 76.00]	63.00 [52.00, 75.00]	65.00 [54.00, 76.00]	60.00 [48.00, 73.00]	<0.001
Gender (%)									0.004
Male	11,615 (58.5)	2,237 (59.4)	4,770 (59.2)	525 (54.9)	1,048 (55.0)	134 (60.9)	2,720 (58.5)	181 (57.1)	
Female	8,254 (41.5)	1,526 (40.6)	3,286 (40.8)	432 (45.1)	859 (45.0)	86 (39.1)	1,929 (41.5)	136 (42.9)	
Ethnicity (%)									<0.001
White	15,320 (77.1)	2,818 (74.9)	6,357 (78.9)	726 (75.9)	1,447 (75.9)	157 (71.4)	3,579 (77.0)	236 (74.4)	
Black	2,013 (10.1)	477 (12.7)	715 (8.9)	99 (10.3)	196 (10.3)	29 (13.2)	461 (9.9)	36 (11.4)	
Others	2,536 (12.8)	468 (12.4)	984 (12.2)	132 (13.8)	264 (13.8)	34 (15.5)	609 (13.1)	45 (14.2)	
Weight, kg	79.90 [67.00, 95.00]	80.00 [66.62, 95.65]	80.00 [67.25, 95.05]	79.40 [64.50, 94.65]	77.90 [65.20, 93.95]	78.20 [64.75, 91.71]	80.00 [67.90, 95.10]	81.50 [67.90, 97.00]	0.001
First_care_unit (%)									<0.001
MICU/SICU	11,048 (55.6)	2,272 (60.4)	4,034 (50.1)	564 (58.9)	1,256 (65.9)	119 (54.1)	2,601 (55.9)	202 (63.7)	
CCU	6,297 (31.7)	938 (24.9)	3,103 (38.5)	257 (26.9)	394 (20.7)	46 (20.9)	1,499 (32.2)	60 (18.9)	
Others	2,524 (12.7)	553 (14.7)	919 (11.4)	136 (14.2)	257 (13.5)	55 (25.0)	549 (11.8)	55 (17.4)	
SOFA	3.00 [2.00, 5.00]	3.00 [2.00, 4.00]	3.00 [2.00, 4.00]	4.00 [2.00, 5.00]	3.00 [2.00, 5.00]	4.00 [3.00, 6.00]	3.00 [2.00, 5.00]	4.00 [3.00, 6.00]	<0.001
Charlson_ comorbidity _index	6.00 [4.00, 8.00]	6.00 [5.00, 8.00]	6.00 [4.00, 8.00]	6.00 [4.00, 8.00]	6.00 [4.00, 8.00]	6.00 [4.00, 8.00]	5.00 [4.00, 8.00]	5.00 [3.00, 8.00]	<0.001
Ventilation (%)									<0.001
No	10,520 (52.9)	2,280 (60.6)	3,921 (48.7)	547 (57.2)	1,096 (57.5)	122 (55.5)	2,372 (51.0)	182 (57.4)	
Yes	9,349 (47.1)	1,483 (39.4)	4,135 (51.3)	410 (42.8)	811 (42.5)	98 (44.5)	2,277 (49.0)	135 (42.6)	
Vasopressor (%)									<0.001
No	13,853 (69.7)	3,176 (84.4)	5,981 (74.2)	458 (47.9)	1,007 (52.8)	76 (34.5)	3,068 (66.0)	87 (27.4)	
Yes	6,016 (30.3)	587 (15.6)	2,075 (25.8)	499 (52.1)	900 (47.2)	144 (65.5)	1,581 (34.0)	230 (72.6)	
CRRT (%)									<0.001
No	19,403 (97.7)	3,694 (98.2)	7,926 (98.4)	928 (97.0)	1,825 (95.7)	207 (94.1)	4,541 (97.7)	282 (89.0)	
Yes	466 (2.3)	69 (1.8)	130 (1.6)	29 (3.0)	82 (4.3)	13 (5.9)	108 (2.3)	35 (11.0)	
AG (mEq/L)	13.00 [11.00, 16.00]	14.00 [12.00, 16.00]	13.00 [11.00, 16.00]	14.00 [11.00, 17.00]	14.00 [12.00, 17.00]	15.00 [13.00, 18.00]	13.00 [11.00, 16.00]	16.00 [13.00, 19.00]	<0.001
Sodium (mEq/L)	139.00 [136.00, 141.00]	139.00 [136.00, 142.00]	139.00 [136.00, 141.00]	138.00 [135.00, 141.00]	138.00 [135.00, 141.00]	139.00 [135.00, 142.00]	138.00 [136.00, 141.00]	137.00 [134.00, 140.00]	<0.001
Potassium (mEq/L)	4.10 [3.80, 4.60]	4.10 [3.80, 4.50]	4.20 [3.80, 4.60]	4.10 [3.80, 4.60]	4.20 [3.70, 4.60]	4.20 [3.70, 4.70]	4.20 [3.80, 4.60]	4.20 [3.70, 4.70]	0.012
Chloride (mEq/L)	106.00 [101.00, 109.00]	105.00 [101.00, 109.00]	106.00 [102.00, 109.00]	106.00 [101.00, 109.00]	105.00 [101.00, 109.00]	105.00 [101.00, 110.00]	105.00 [101.00, 109.00]	105.00 [100.00, 109.00]	<0.001
Bicarbonate (mEq/L)	23.00 [20.00, 26.00]	24.00 [21.00, 26.00]	23.00 [21.00, 26.00]	22.00 [19.00, 25.00]	22.00 [19.00, 25.00]	21.00 [18.00, 24.25]	23.00 [20.00, 26.00]	20.00 [16.00, 24.00]	<0.001
Phosphate (mEq/L)	3.40 [2.70, 4.30]	3.50 [2.70, 4.30]	3.40 [2.70, 4.20]	3.50 [2.70, 4.50]	3.50 [2.70, 4.50]	3.90 [3.00, 5.03]	3.40 [2.70, 4.30]	3.70 [2.90, 4.90]	<0.001
Glucose (mg/dL)	125.00 [104.00, 158.00]	129.00 [106.00, 165.00]	124.00 [104.00, 155.00]	126.00 [103.00, 165.00]	124.00 [102.00, 157.00]	146.00 [113.75, 197.25]	124.00 [103.00, 155.00]	129.00 [105.00, 178.00]	<0.001
Creatinine (mg/dL)	1.00 [0.70, 1.70]	1.10 [0.80, 1.80]	1.00 [0.70, 1.60]	1.10 [0.80, 1.70]	1.10 [0.70, 1.80]	1.30 [0.90, 2.02]	1.00 [0.70, 1.60]	1.30 [0.80, 2.40]	<0.001
BUN (mg/dL)	21.00 [14.00, 35.00]	22.00 [15.00, 38.00]	20.00 [14.00, 33.00]	22.00 [15.00, 35.00]	22.00 [14.00, 39.00]	23.00 [16.00, 40.25]	21.00 [14.00, 34.00]	24.00 [15.00, 42.00]	<0.001
RBC (m/μL)	3.38 [2.97, 3.83]	3.43 [3.03, 3.91]	3.38 [2.98, 3.82]	3.35 [2.89, 3.81]	3.35 [2.89, 3.82]	3.30 [2.85, 3.90]	3.35 [2.94, 3.79]	3.50 [3.02, 4.04]	<0.001
WBC (k/μL)	11.40 [8.00, 15.70]	10.60 [7.70, 14.50]	11.40 [8.20, 15.33]	12.00 [8.70, 17.40]	11.90 [7.75, 17.20]	12.75 [8.28, 18.12]	11.80 [8.30, 16.20]	11.80 [6.90, 18.10]	<0.001
Platelet (k/μL)	176.00 [124.00, 247.00]	182.00 [130.00, 249.00]	173.00 [122.00, 240.00]	178.00 [119.00, 254.00]	177.00 [122.00, 256.00]	174.50 [114.00, 282.00]	177.00 [123.00, 250.00]	173.00 [112.00, 252.00]	<0.001
RDW (%)	14.90 [13.80, 16.50]	14.80 [13.70, 16.30]	14.70 [13.70, 16.30]	15.10 [13.90, 16.60]	15.30 [14.10, 17.10]	15.30 [14.10, 16.90]	14.90 [13.80, 16.60]	15.10 [14.00, 16.90]	<0.001
Hemoglobin (g/dL)	10.10 [8.90, 11.50]	10.30 [9.10, 11.70]	10.10 [8.90, 11.50]	10.00 [8.70, 11.30]	10.00 [8.70, 11.40]	9.90 [8.50, 11.30]	10.00 [8.80, 11.30]	10.60 [9.10, 11.90]	<0.001
pCO2 (mmHg)	40.00 [35.00, 46.00]	40.00 [35.00, 46.00]	40.00 [35.00, 46.00]	40.00 [35.00, 47.00]	40.00 [34.00, 46.00]	42.00 [36.00, 49.00]	40.00 [35.00, 46.00]	39.00 [32.00, 46.00]	0.001
pO2 (mmHg)	107.00 [72.00, 162.00]	106.00 [73.00, 160.50]	111.00 [76.00, 167.00]	109.00 [73.00, 162.00]	98.00 [65.00, 153.50]	102.50 [67.75, 184.50]	105.00 [70.00, 159.00]	94.00 [66.00, 150.00]	<0.001
pH	7.37 [7.32, 7.43]	7.39 [7.34, 7.44]	7.38 [7.32, 7.43]	7.36 [7.29, 7.42]	7.36 [7.30, 7.42]	7.31 [7.22, 7.39]	7.37 [7.31, 7.42]	7.33 [7.26, 7.40]	<0.001
Shock_index1 (min^–1^.mmHg^–1^)	0.88 [0.73, 1.06]	0.62 [0.54, 0.70]	0.84 [0.75, 0.94]	1.33 [1.22, 1.47]	1.20 [1.08, 1.33]	1.86 [1.70, 2.09]	0.97 [0.88, 1.07]	1.45 [1.27, 1.59]	<0.001
Shock_index2 (min^–1^.mmHg^–1^)	0.85 [0.71, 1.02]	0.60 [0.54, 0.67]	0.80 [0.73, 0.88]	1.12 [0.99, 1.24]	1.20 [1.10, 1.32]	1.37 [1.17, 1.66]	0.97 [0.89, 1.06]	1.49 [1.35, 1.67]	<0.001
Shock_index3 (min^–1^.mmHg^–1^)	0.84 [0.70, 1.00]	0.60 [0.53, 0.65]	0.79 [0.72, 0.86]	0.96 [0.85, 1.07]	1.21 [1.11, 1.32]	1.17 [1.03, 1.32]	0.98 [0.90, 1.07]	1.51 [1.37, 1.67]	<0.001
Shock_index4 (min^–1^.mmHg^–1^)	0.83 [0.69, 0.99]	0.59 [0.53, 0.65]	0.78 [0.71, 0.85]	0.89 [0.79, 0.97]	1.20 [1.11, 1.32]	1.07 [0.94, 1.19]	0.98 [0.91, 1.07]	1.51 [1.40, 1.70]	<0.001
Shock_index5 (min^–1^.mmHg^–1^)	0.82 [0.69, 0.98]	0.59 [0.53, 0.66]	0.77 [0.70, 0.84]	0.84 [0.76, 0.93]	1.19 [1.10, 1.31]	1.06 [0.90, 1.18]	0.98 [0.90, 1.06]	1.47 [1.36, 1.66]	<0.001
Shock_index6 (min^–1^.mmHg^–1^)	0.81 [0.68, 0.97]	0.60 [0.53, 0.67]	0.76 [0.69, 0.84]	0.83 [0.74, 0.93]	1.16 [1.05, 1.29]	1.05 [0.90, 1.18]	0.96 [0.88, 1.06]	1.41 [1.25, 1.61]	<0.001
Ventilation-free days in 28 days, day	26.54 [24.67, 27.50]	26.83 [25.08, 27.79]	26.71 [25.12, 27.50]	26.21 [24.00, 27.38]	26.04 [23.50, 27.33]	25.38 [22.01, 26.71]	26.42 [24.42, 27.46]	25.54 [21.29, 27.25]	<0.001
Vasopressor-free days in 28 days, day	28.00 [27.38, 28.00]	28.00 [28.00, 28.00]	28.00 [27.71, 28.00]	27.79 [26.62, 28.00]	27.79 [26.19, 28.00]	27.02 [25.49, 28.00]	28.00 [27.17, 28.00]	26.75 [24.75, 27.92]	<0.001
Death in 28 days (%)									
No	17,019 (85.7)	3,315 (88.1)	7,205 (89.4)	800 (83.6)	1,414 (74.1)	150 (68.2)	3,937 (84.7)	198 (62.5)	
Yes	2,850 (14.3)	448 (11.9)	851 (10.6)	157 (16.4)	493 (25.9)	70 (31.8)	712 (15.3)	119 (37.5)	
Death in hospital (%)									<0.001
No	16,811 (84.6)	3,289 (87.4)	7,137 (88.6)	786 (82.1)	1,380 (72.4)	148 (67.3)	3,882 (83.5)	189 (59.6)	
Yes	3,058 (15.4)	474 (12.6)	919 (11.4)	171 (17.9)	527 (27.6)	72 (32.7)	767 (16.5)	128 (40.4)	
Death in ICU (%)									<0.001
No	17,791 (89.5)	3,457 (91.9)	7,446 (92.4)	837 (87.5)	1,530 (80.2)	160 (72.7)	4,148 (89.2)	213 (67.2)	
Yes	2,078 (10.5)	306 (8.1)	610 (7.6)	120 (12.5)	377 (19.8)	60 (27.3)	501 (10.8)	104 (32.8)	
Length of stay in hospital, day	7.83 [4.96, 14.04]	7.96 [5.04, 14.10]	7.04 [4.79, 12.58]	8.75 [5.46, 14.96]	9.71 [5.58, 17.38]	9.60 [5.43, 18.11]	8.17 [5.04, 14.46]	10.88 [3.58, 21.38]	<0.001
Length of stay in ICU, day	3.21 [1.88, 6.46]	3.29 [1.83, 6.79]	2.92 [1.71, 5.38]	3.67 [2.08, 7.08]	4.21 [2.50, 8.00]	5.02 [2.61, 8.90]	3.17 [1.92, 6.75]	5.04 [2.08, 11.17]	<0.001

MICU, medical intensive care unit; SICU, surgical intensive care unit; CCU, coronary care unit; SOFA, Sequential Organ Failure Assessment; CRRT, continuous renal replacement therapy; AG, anion gap; BUN, blood urea nitrogen; RBC, red blood cells; WBC, white blood cells; RDW, red blood cell distribution width.

### Prognostic value of shock index

**FIGURE 2:**
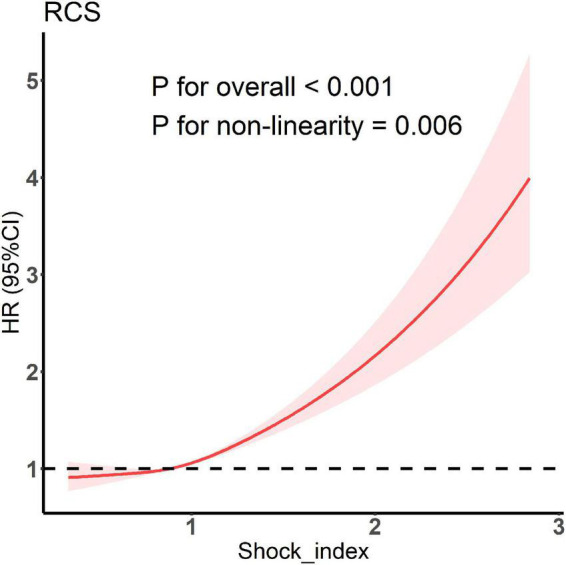
Restricted cubic spline model showing association between shock index and the occurrence of in-hospital death.

The C-index of the model with both SOFA and initial SI was significantly higher than that of the model with SOFA only (0.599 [0.587–0.611] vs. 0.579 [0.567–0.590]; *P* < 0.001). Thus, SI was found to have a predictive value for the risk of in-hospital death independent of the SOFA score. The results of RCS showed a non-linear relationship between SI and the risk of in-hospital death (Figure 2). As SI increased, in-hospital death risk increased, and the slope gradually increased. Therefore, SI was a risk factor for in-hospital death. As SI increased, its effect on in-hospital death risk also increased.

### Shock index trajectory classification

The goodness-of-fit statistics of the LGMM models are shown in Table 2. As the number of classes increased, the log-likelihood ratio showed a rising trend, whereas the information criterion indexes showed a decreasing trend. These results indicated that the fitting effect became better as the number of classes increased. The sample size of the model with eight classes was less than 1% of the total population, resulting in insufficient performance of subsequent statistical analyses. By contrast, the model with seven classes met this criteria. Moreover, the entropy of seven classes was higher than that of the entropy with six and eight classes, both of which was also higher than 0.7. As shown in Supplementary Table 1, the posterior probability of each class of the model with seven classes was higher than 70%. Therefore, we finally selected the model with seven classes as the best model.

**TABLE 2:** Statistics for choosing the best number of classes.

Number of classes	Log likelihood	AIC	SABIC	Entropy	%Class 1	%Class 2	%Class 3	%Class 4	%Class 5	%Class 6	%Class 7	%Class 8
1	−4563.9	9135.8	9154.7	1.0000000	100.00000							
2	17710.2	–35404.3	–35366.6	0.8694703	68.56409	31.43591						
3	26406.2	–52788.3	–52731.7	0.8479593	39.90639	47.27968	12.81393					
4	29951.7	–59871.4	–59795.9	0.8346446	25.48694	46.17746	22.74397	5.59163				
5	32385.2	–64730.3	–64635.9	0.8578074	21.76254	24.74206	2.67754	45.42252	5.39534			
6	34037.1	–68026.1	–67912.9	0.8416932	18.42066	40.40465	2.73290	9.55257	26.92637	1.96286		
7	35030.6	–70005.1	–69873.0	0.8453256	18.93905	40.54557	4.81655	9.59787	1.10725	23.39826	1.59545	
8	35965.5	–71867.0	–71716.0	0.8238116	12.95485	4.95244	32.65388	30.27832	11.98853	0.65932	0.87574	5.63692

AIC, Akaike information criterion; BIC, Bayesian information criteria; SABIC, sample-adjusted information criteria.

**FIGURE 3:**
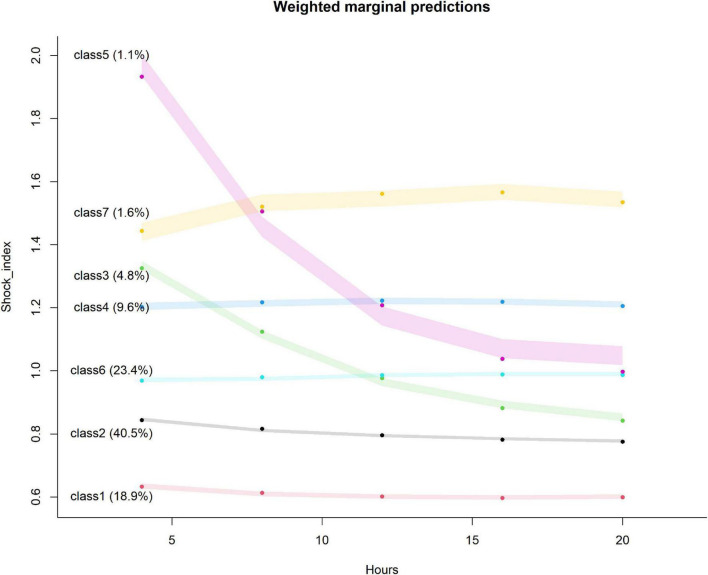
Seven classes identified by trajectories of shock index. The shaded area indicates the 95% confidence interval for each mean trajectory. The percentages in the parentheses indicate the percentages of patients each class accounts for.

**FIGURE 4:**
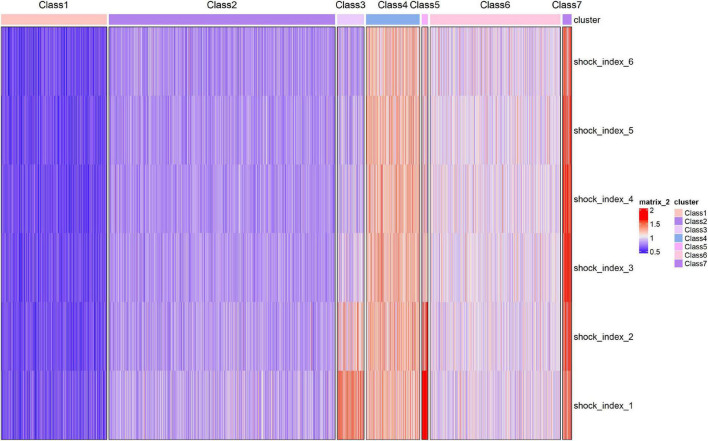
Heatmap of shock index over time for the seven identified classes. Each patient is represented by a single line colored.

The trajectory of the seven trajectory classes are shown in Figure 3. Classes 1 and 2 were at the level of 0.6 and 0.8, respectively, with a slight downward trend. The SI values of classes 4, 6, and 7 remained at the positions of 1.2, 1.0, and 1.4, respectively, and showed a slight upward trend. The initial SI level of class 3 was relatively high, between 1.2 and 1.4, and the rate of decline was relatively fast. The initial SI value of class 5 was the highest, close to 2.0, and the decline was the fastest. It finally dropped to about 1.0. The formulae of the model with seven classes are provided in Supplementary Table 2. The pattern of SI over time for each patient class was described in the heat map (Figure 4).

The characteristics of the patients in each trajectory class are given in Table 1. The higher the initial SI was, the higher the proportion of ventilator, vasopressor, and CRRT use was.

### Predictive value of shock index trajectory to primary outcome

#### Cox proportional hazard model

The results of the five Cox proportional hazard models adjusted for different numbers of covariates are shown in Table 3. In the first four models, no significant increase in the risk of in-hospital death was found in class 2 with the reference group class 1, but a significant increase in risk was observed from class 3 to class 7. In model 5, after the additional adjustment of treatment measures, no significant increase in risk was observed in class 3 compared with that in class 1. The risk of classes 4, 5, 6, and 7 increased by 1.741 (1.529–1.982), 1.946 (1.510–2.509), 1.312 (1.166–1.475), and 2.610 (2.130–3.199), respectively. The results of model 5 with different reference class sets are shown in Supplementary Table 3.

**TABLE 3:** Results of Cox proportional hazard models.

Class	Model 1		Model 2		Model 3		Model 4		Model 5	
	HR (95%CI)	*P*-value	HR (96%CI)	*P*-value	HR (96%CI)	*P*-value	HR (97%CI)	*P*-value	HR (97%CI)	*P*-value
Class 1	Reference		Reference		Reference		Reference		Reference	
Class 2	0.977 (0.874–1.091)	0.676	1.036 (0.927–1.158)	0.528	1.032 (0.923–1.154)	0.579	1.032 (0.923–1.154)	0.578	1.009 (0.902–1.129)	0.873
Class 3	1.289 (1.082–1.536)	0.005	1.342 (1.126–1.600)	0.001	1.320 (1.107–1.574)	0.002	1.266 (1.060–1.511)	0.009	1.168 (0.976–1.397)	0.091
Class 4	1.859 (1.642–2.105)	<0.001	2.103 (1.855–2.383)	<0.001	2.013 (1.776–2.281)	<0.001	1.875 (1.651–2.129)	<0.001	1.741 (1.529–1.982)	<0.001
Class 5	2.238 (1.746–2.867)	<0.001	2.648 (2.065–3.395)	<0.001	2.376 (1.852–3.049)	<0.001	2.132 (1.658–2.742)	<0.001	1.946 (1.510–2.509)	<0.001
Class 6	1.274 (1.136–1.429)	<0.001	1.425 (1.270–1.599)	<0.001	1.401 (1.248–1.572)	<0.001	1.370 (1.220–1.539)	<0.001	1.312 (1.166–1.475)	<0.001
Class 7	2.540 (2.088–3.089)	<0.001	3.345 (2.746–4.074)	<0.001	3.176 (2.607–3.870)	<0.001	2.929 (2.399–3.577)	<0.001	2.610 (2.130–3.199)	<0.001

HR, hazard ratio; CI, confidence interval. No covariables were adjusted in Model 1; Model 2 adjusted age, gender, ethnicity, weight and first_care_unit; Model 3 adjusted the covariables in Model 2, as well as SOFA and Charlson_comorbidity_index; Model 4 adjusted the covariables in Model 3, as well as AG, sodium, potassium, chloride, bicarbonate, phosphate, glucose, creatinine, BUN, RBC, WBC, platelet, RDW, hemoglobin, pH, pCO2, and pO2; Model 5 adjusted the covariables in Model 4, as well as ventilator, CRRT and vasopressor.

#### Double robust analysis

The baseline characteristics of the patients in the IPTW cohort are provided in Supplementary Table 4. The SMDs of the covariables of the original cohort and the IPTW cohort are given in Supplementary Figure 2. After IPTW, the unbalance of covariables between classes substantially decreased. Except for ethnicity, first_care_unit, Charlson_comorbidity_index, ventilator use, and vasopressor use, all the other covariables reached the balance between classes. The relative effects of covariables on the GBM model are shown in Supplementary Figure 3. The top five variables were WBC, weight, platelet, glucose, and pO2. The degree of relative effect of the covariates may reflect the degree of their influence on SI trajectory. The results of the double robust analysis are listed in Supplementary Table 5, which were similar to the results generated in the original cohort, indicating the stability of the research results.

#### C-index

The C-index of the model containing both SOFA and trajectory class was 0.628 (0.616–0.640), which was substantially higher than that of the model containing only SOFA and the model containing both SOFA and the initial SI. This result was obtained because the SI trajectory class considered both the initial level and the change trend of SI.

#### Subgroup analysis

The results of subgroup analysis are summarized in Supplementary Table 6. The influence of SI trajectory on the risk of in-hospital death was different in subgroups with different degrees of Charlson_comorbidity_index. Specifically, the effect was greater in people with more comorbidities.

### Predictive value of shock index trajectory to secondary outcomes

The influence of trajectory class on secondary outcomes is shown in Supplementary Table 7. No significant difference was observed between classes 2 and 3 in the risk of in-ICU death or 28-day death compared with that of class 1. The in-ICU death risk of classes 4, 5, 6, and 7 was 1.714 (1.464–2.007), 1.945 (1.462–2.589), 1.236 (1.068–1.431), and 2.361 (1.868–2.983) times higher than that of class 1; moreover, their 28-day death risk increased by 1.732 (1.516–1.980), 1.934 (1.494–2.503), 1.287 (1.140–1.453), and 2.655 (2.152–3.277), respectively. No significant difference in ventilation-free days in 28 days was observed in classes 2 and 3 compared with that in class 1. Compared with those in class 1, the ventilation-free days in 28 days of classes 4, 5, 6, and 7 decreased by 0.529 (0.729–0.329), 0.972 (1.457–0.487), 0.254 (0.409–0.099), and 1.190 (1.603–0.778) days, respectively. Compared with those of class 1, the vasopressor-free days of classes 2, 3, 4, 5, 6, and 7 decreased by 0.156 (0.234–0.078), 0.461 (0.603–0.319), 0.844 (0.955–0.734), 0.967 (1.239–0.696), 0.447 (0.534–0.360), and 1.602 (1.831–1.372), respectively.

## Discussion

Shock index is a classic indicator of shock that is more sensitive than traditional vital signs (19, 20). Several published studies have been conducted on the prognostic role of SI in predicting clinical outcomes of patients. For example, a recent prospective study showed that SI can predict the risk of death in patients with sepsis (21). Charles et al. revealed that elevated SI in patients with severe sepsis in the emergency room may be an important indicator of disease escalation and risk of cardiovascular failure (22). However, scholars have mostly studied the prognostic value of static SI indicators, and we hope that by exploring the clinical significance hidden behind the change trend of SI over time, clinicians can further understand the severity of the disease of patients, which can help them make timely and effective clinical decisions and improve the prognosis of patients.

In the present study, we first conducted a cross-sectional analysis of SI. We found that the C-index of the SOFA combined with the initial SI model was substantially higher than that of the SOFA model alone, indicating that SI has a predictive value for the risk of in-hospital death independent of the SOFA score. Moreover, the results of RCS established a non-linear relationship between SI and the risk of in-hospital death. As SI increased, its influence on the risk of in-hospital death became greater, consistent with the results of previous studies (23). We then studied the longitudinal trajectory of SI in patients with sepsis within 24 h after diagnosis. The LGMM model divided the patients into seven classes of different trajectories. The C-index of the model containing both SOFA trajectory class was 0.628 (0.616–0.640), which was considerably higher than that of the model containing SOFA alone and the model containing both SOFA and initial SI. This result further illustrated that the trajectory of SI change has an important value for the prognosis of patients with sepsis. Cox analysis revealed that compared with the patients in class 1, the patients in classes 4, 5, 6, and 7 had an increased risk of in-hospital death. We will explain these results step by step.

The SI of the patients in classes 1 and 2 was continuously at a low level of 0.62 and 0.84, respectively. Previous studies have shown that the normal range of SI is 0.5–0.7 (22),and some scholars reported that the prognosis of severely ill patients is worse when SI > 0.9 (24). Therefore, the SI of these two classes of patients was close to the normal level, and thus it had no notable effect on the prognosis. Although the initial level of the patients in class 3 was 1.33, which was relatively high, the trend showed a decline and eventually dropped to 0.83. Studies have shown that when the SI is close to 1.0, it often indicates that the patient’s hemodynamic status is worsening and shock may occur (25). However, when the SI is decreasing, meaning that the patient is being treated aggressively and effectively, possibly with positive inotropic drugs, like dobutamine. Thus, the risk of in-hospital death for this class of patients is also relatively small. The patients in classes 4, 6, and 7 had been at a relatively high level of 1.20, 0.97, and 1.45, respectively, and the risk can be ranked as class 6 < class 4 < class 7. An increase in SI indicates an increase in heart rate and a decrease in systolic blood pressure (26), suggesting that the patient has acute hypovolemia and circulatory failure (27), all of which will increase the patient’s risk of death. The initial level of the patients in class 5 was at a relatively high stage of 1.86, indicating that the patients had severe circulatory failure at the beginning (28). Although the trend decreased in the later stage, the prognosis remained poor. The cause was severe circulatory failure in the early stage, tissue ischemia, hypoxia (29), neurohumoral factor imbalance (30), insufficient microcirculation of important organs (31), metabolic disorders (32), and systemic organ dysfunction (33). Although the trend dropped in the later period, the prognosis of patients remained owing to the inability to recover from organ damage.

We also noticed an interesting phenomenon. We adjusted ventilator, vasopressor, and CRRT intervention methods in model 5 and found that the risk of each group was lower than that of model 4, indicating that the aforementioned intervention methods are risk factors for patients. The patient required external intervention due to severe disease and lung, circulation, and kidney dysfunction. Hence, the prognosis was poor. Moreover, as can be observed from the baseline table, the SI trend was different, and the percentages of the three intervention methods were different. All secondary outcomes in our study also presented similar results to in-hospital death. Therefore, in clinical practice, it is worthwhile to pay attention to trends in SI and adopt them to further guide external treatments and interventions for patients, which can help improve their prognosis. To illustrate, when a patient is treated with positive inotropic medications, changes in SI can reflect whether the treatment is effective and can guide clinicians to avoid several of the above-mentioned trends that can increase the patient’s risk of death.

Subgroup analysis revealed that in people with a higher Chalson comorbidity index, SI had a greater impact on sepsis. Chalson comorbidity index can predict the prognosis of patients with sepsis (34, 35) as it reflects the basic state of patients to a certain extent. The higher the index, the worse the basic state of the patient will be. When sepsis occurs, the stress compensation ability of each organ is weak. Thus, SI has a greater impact on such patients.

### Strengths and Limitations

The strengths of this study are as follows. First, MIMIC-IV has a large sample size, and its data are of high quality and newer than those of MIMIC-III. Second, the prognostic value of SI in patients with sepsis was explored from two aspects, namely, cross-section analysis and longitudinal trajectory analysis. Finally, Cox proportional risk model was used and double robust analysis and subgroup analysis were performed to validate the stability of the results comprehensively and explore the potential correction effect.

Inevitably, the present study also has several limitations. First, the data of MIMIC-IV were from a single center, and thus the extrapolation of the results will be limited. Moreover, the conclusions must be verified via prospective multiple center studies. Second, the missing values of covariables were imputated, and the research results had information bias to some extent. Third, some potential confounding factors such as C-reactive protein and procalcitonin were not taken into account due to the high data missing rate. Finally, the relative effects of covariables in the GBM model reflected the degree of covariable imbalance among trajectory classes, but the influence and mechanism of these covariables on trajectory classes should be explored further in subsequent studies.

## Conclusion

Both the initial SI and the trajectory of SI were found to be independent factors that affect the prognosis of patients with sepsis. In the cross-sectional analysis, as the SI increased, its impact on the risk of in-hospital death became greater. In terms of longitudinal trajectory, compared with the SI continuously at a low level of 0.6, the SI continued to be at a level higher than 1.0, and the patients in the class whose initial SI was at a high level of 1.2 and then declined had a worse prognosis. Therefore, in clinical treatment, we should closely monitor the basic vital signs of patients and arrive at appropriate clinical decisions on basis of their change trajectory to improve their prognosis and increase their survival rate.

## Data availability statement

The datasets presented in this study can be found in online repositories. The names of the repository/repositories and accession number(s) can be found below: https://physionet.org/content/mimiciv/1.0/.

## Author contributions

FX created the study protocol, performed the statistical analyses, and wrote the manuscript draft. LZ wrote the manuscript draft. TH, DH, and RY assisted with the study design and collected the data. SZ, AF, and LH checked the integrity of the data and the accuracy of the data analysis. HY and JL assisted with the study design and the manuscript revision. All authors read and approved the final manuscript.

## Supplementary material

Supplementary Figure 1 Data missing before multiple imputation. SOFA, Sequential Organ Failure Assessment; CRRT, continuous renal replacement therapy; AG, anion gap; BUN, blood urea nitrogen; RBC, red blood cells; WBC, white blood cells; RDW, red blood cell distribution width.

Supplementary Figure 2 SMD of covariable before and after IPTW. IPTW, inverse probability of treatment weighting; SOFA, Sequential Organ Failure Assessment; CRRT, continuous renal replacement therapy; AG, anion gap; BUN, blood urea nitrogen; RBC, red blood cells; WBC, white blood cells; RDW, red blood cell distribution width.

Supplementary Figure 3 The contribution of each covariate to the GBM model. SOFA, Sequential Organ Failure Assessment; CRRT, continuous renal replacement therapy; AG, anion gap; BUN, blood urea nitrogen; RBC, red blood cells; WBC, white blood cells; RDW, red blood cell distribution width.
