# Supplementary figures and images for: Effects of growth trajectory of shock index within 24 h on the prognosis of patients with sepsis
Source: Front Med (Lausanne). 2022 Aug 22;9:898424. doi: 10.3389/fmed.2022.898424 (PMC9441919; doi:10.3389/fmed.2022.898424)

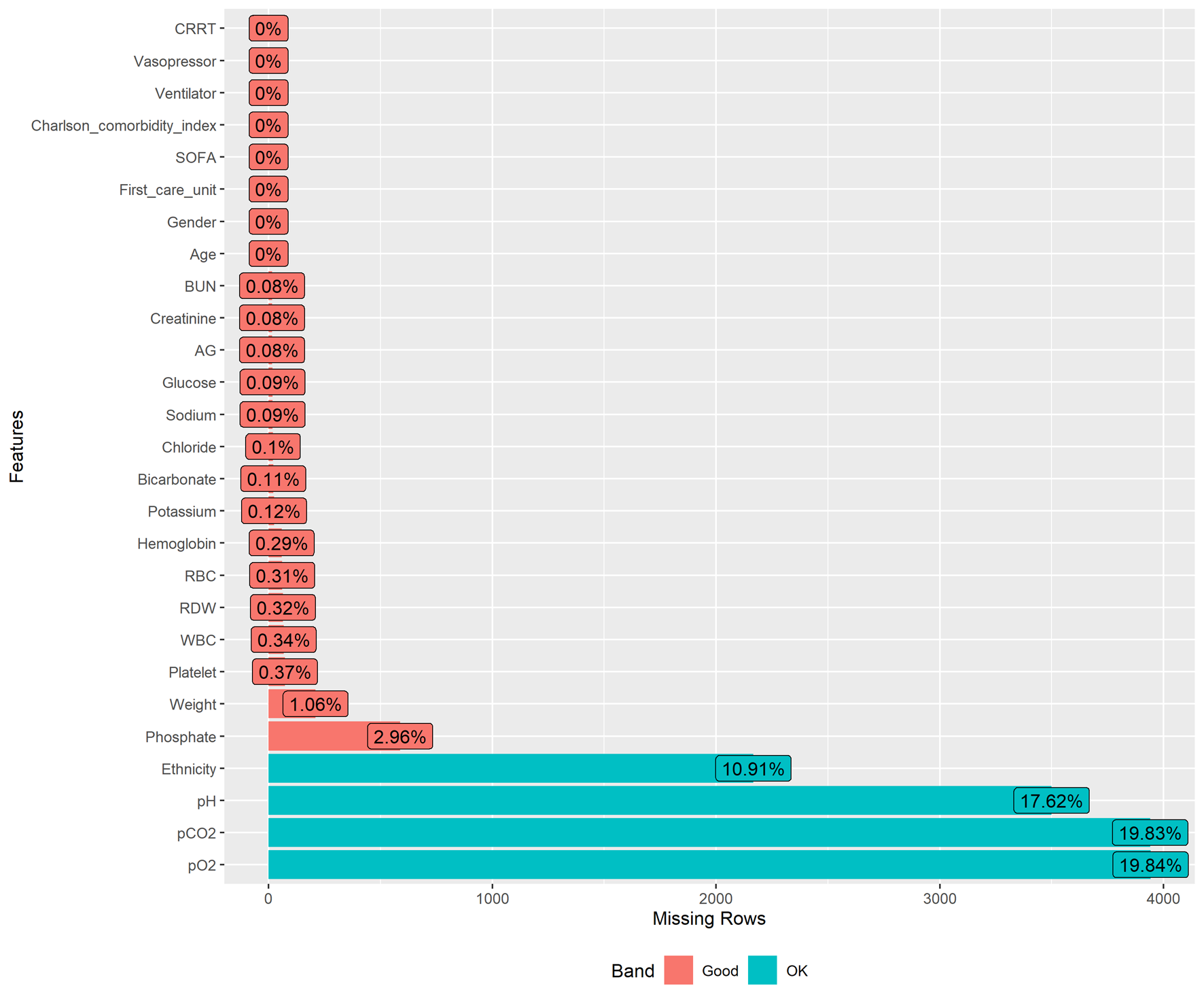

Supplement: Supplementary Figure 1 — Data missing before multiple imputation. SOFA, Sequential Organ Failure Assessment; CRRT, continuous renal replacement therapy; AG, anion gap; BUN, blood urea nitrogen; RBC, red blood cells; WBC, white blood cells; RDW, red blood cell distribution width. [file Image_1.TIFF]

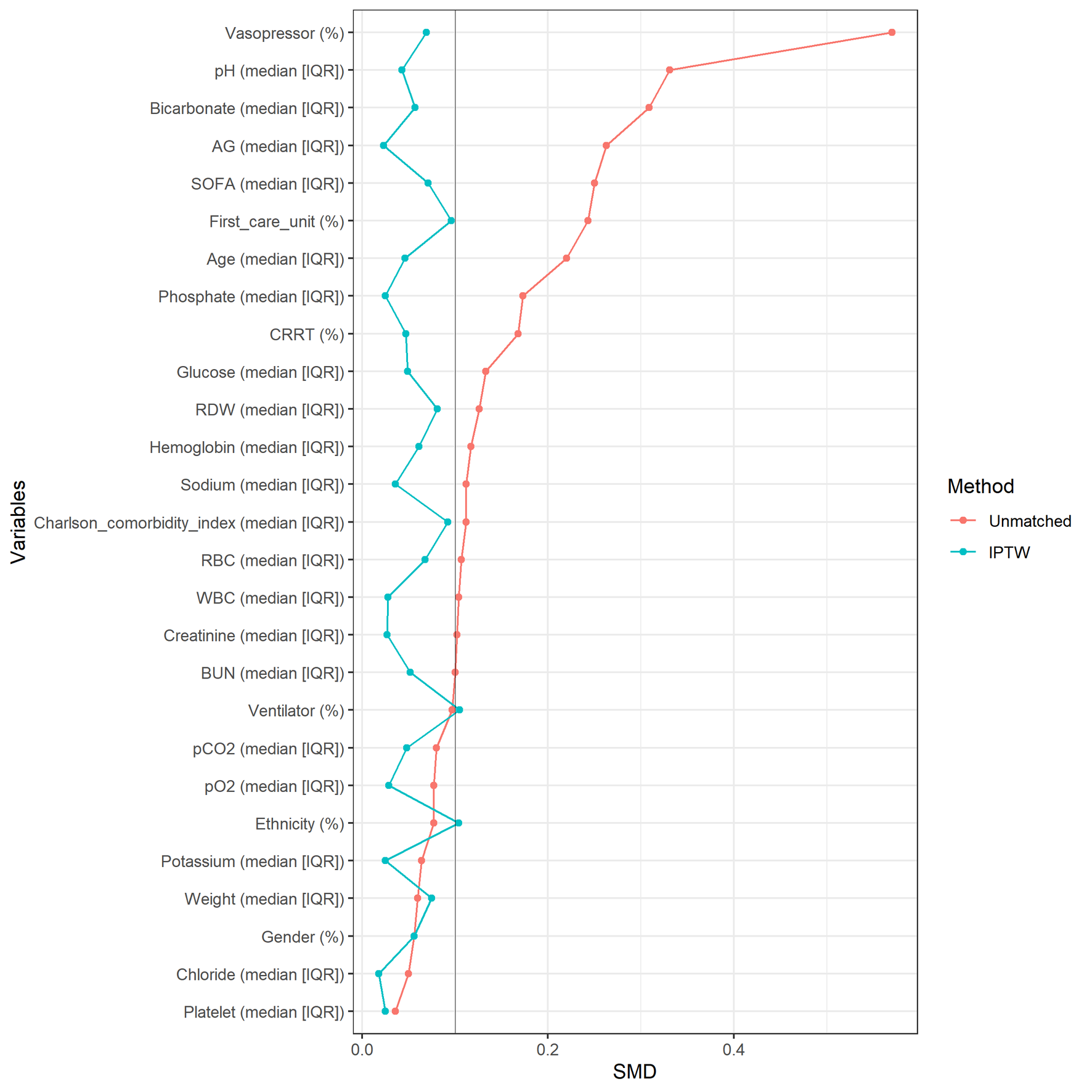

Supplement: Supplementary Figure 2 — SMD of covariable before and after IPTW. IPTW, inverse probability of treatment weighting; SOFA, Sequential Organ Failure Assessment; CRRT, continuous renal replacement therapy; AG, anion gap; BUN, blood urea nitrogen; RBC, red blood cells; WBC, white blood cells; RDW, red blood cell distribution width. [file Image_2.TIFF]

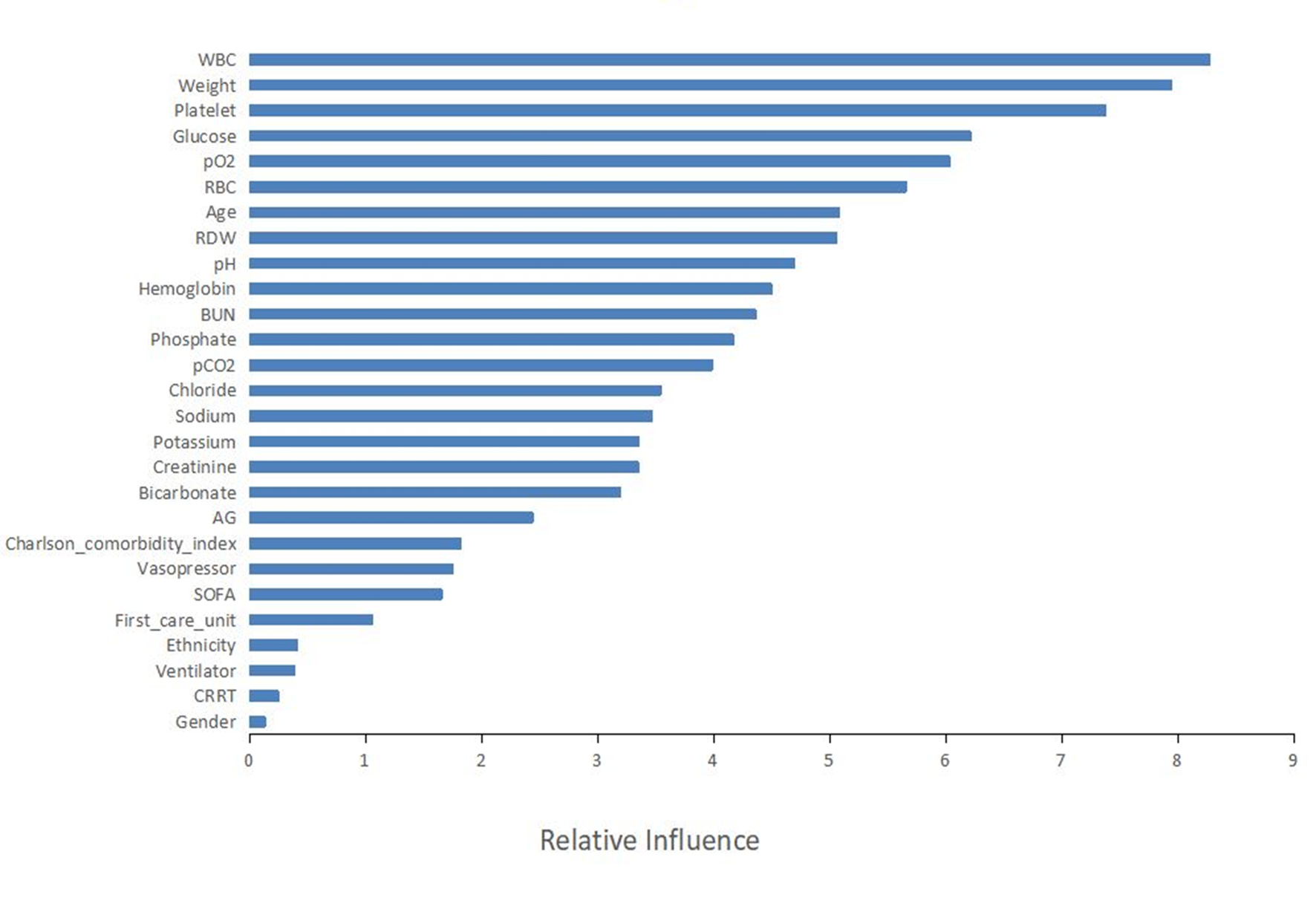

Supplement: Supplementary Figure 3 — The contribution of each covariate to the GBM model. SOFA, Sequential Organ Failure Assessment; CRRT, continuous renal replacement therapy; AG, anion gap; BUN, blood urea nitrogen; RBC, red blood cells; WBC, white blood cells; RDW, red blood cell distribution width. [file Image_3.TIF]
