# Supplementary material for: Effects of growth trajectory of shock index within 24 h on the prognosis of patients with sepsis
Source: Front Med (Lausanne). 2022 Aug 22;9:898424. doi: 10.3389/fmed.2022.898424 (PMC9441919; doi:10.3389/fmed.2022.898424)
Supplement: Supplementary file 4 [file Data_Sheet_1.docx]

Table S1. Mean of posterior probabilities in each class.

| **Class** | **probability1** | **probability2** | **probability3** | **probability4** | **probability5** | **probability6** | **probability7** |
| --- | --- | --- | --- | --- | --- | --- | --- |
| **class1** | 90.23% | 9.77% | 0.00% | 0.00% | 0.00% | 0.00% | 0.00% |
| **class2** | 5.50% | 87.88% | 0.76% | 0.00% | 0.00% | 5.86% | 0.00% |
| **class3** | 0.00% | 4.25% | 80.90% | 2.47% | 0.82% | 11.55% | 0.00% |
| **class4** | 0.00% | 0.00% | 1.40% | 90.00% | 0.48% | 6.72% | 1.40% |
| **class5** | 0.00% | 0.00% | 3.02% | 4.09% | 92.01% | 0.00% | 0.87% |
| **class6** | 0.00% | 8.73% | 3.78% | 3.11% | 0.00% | 84.38% | 0.00% |
| **class7** | 0.00% | 0.00% | 0.00% | 4.99% | 0.71% | 0.00% | 94.30% |

Table S2. Fixed effects in the longitudinal seven classes model.

| **Item** | **Coefficient** | **Standard error** | **Wald statistic** | **P-value** |
| --- | --- | --- | --- | --- |
| **Intercept class1** | 0.65933 | 0.00506 | 130.422 | <0.001 |
| **Intercept class2** | 0.87726 | 0.00399 | 219.710 | <0.001 |
| **Intercept class3** | 1.58066 | 0.01879 | 84.111 | <0.001 |
| **Intercept class4** | 1.17768 | 0.00955 | 123.355 | <0.001 |
| **Intercept class5** | 2.48894 | 0.03978 | 62.569 | <0.001 |
| **Intercept class6** | 0.95362 | 0.00570 | 167.252 | <0.001 |
| **Intercept class7** | 1.33089 | 0.01778 | 74.869 | <0.001 |
| **poly1 class1** | -0.03020 | 0.00295 | -10.232 | <0.001 |
| **poly1 class2** | -0.03752 | 0.00219 | -17.159 | <0.001 |
| **poly1 class3** | -0.28185 | 0.01048 | -26.896 | <0.001 |
| **poly1 class4** | 0.02949 | 0.00486 | 6.064 | <0.001 |
| **poly1 class5** | -0.62009 | 0.02371 | -26.151 | <0.001 |
| **poly1 class6** | 0.01722 | 0.00337 | 5.106 | <0.001 |
| **poly1 class7** | 0.13097 | 0.01271 | 10.302 | <0.001 |
| **poly2 class1** | 0.00364 | 0.00041 | 8.896 | <0.001 |
| **poly2 class2** | 0.00341 | 0.00030 | 11.413 | <0.001 |
| **poly2 class3** | 0.02682 | 0.00130 | 20.587 | <0.001 |
| **poly2 class4** | -0.00480 | 0.00066 | -7.292 | <0.001 |
| **poly2 class5** | 0.06433 | 0.00300 | 21.457 | <0.001 |
| **poly2 class6** | -0.00212 | 0.00043 | -4.891 | <0.001 |
| **poly2 class7** | -0.01803 | 0.00175 | -10.283 | <0.001 |

Table S3. The hazard ratio for each two classes in model5.

| **Treat**  **Control** | **class1** | **class2** | **class3** | **class4** | **class5** | **class6** |
| --- | --- | --- | --- | --- | --- | --- |
| **class2** | 1.009(0.902-1.129) |  |  |  |  |  |
| **class3** | 1.168(0.976-1.397) | 1.157(0.980-1.366) |  |  |  |  |
| **class4** | 1.741(1.529-1.982) | 1.725(1.545-1.926) | 1.491(1.253-1.774) |  |  |  |
| **class5** | 1.946(1.510-2.509) | 1.929(1.512-2.460) | 1.667(1.264-2.200) | 1.118(0.872-1.433) |  |  |
| **class6** | 1.312(1.166-1.475) | 1.300(1.180-1.432) | 1.123(0.950-1.328) | 0.753(0.674-0.843) | 0.674(0.528-0.860) |  |
| **class7** | 2.610(2.130-3.199) | 2.586(2.137-3.129) | 2.235(1.773-2.818) | 1.499(1.233-1.822) | 1.341(1.003-1.793) | 1.990(1.644-2.408) |

Table S4. Baseline characteristics of seven classes afer IPTW.

| **Variable** | **Overall** | **Class1** | **Class2** | **Class3** | **Class4** | **Class5** | **Class6** | **P-value** |
| --- | --- | --- | --- | --- | --- | --- | --- | --- |
| **N** | 3425.18 | 7895.97 | 846.86 | 1705.76 | 142.83 | 4499.91 | 216.84 |  |
| **Age,year** | 66.94 (15.57) | 66.19 (15.56) | 66.03 (15.70) | 65.42 (16.05) | 64.97 (16.27) | 65.63 (15.71) | 63.78 (15.94) | 0.002 |
| **Gender(%)** |  |  |  |  |  |  |  | 0.237 |
| **Male** | 2028.0 (59.2) | 4630.8 (58.6) | 474.5 (56.0) | 960.2 (56.3) | 83.2 (58.3) | 2650.1 (58.9) | 114.2 (52.7) |  |
| **Female** | 1397.2 (40.8) | 3265.2 (41.4) | 372.4 (44.0) | 745.6 (43.7) | 59.6 (41.7) | 1849.8 (41.1) | 102.6 (47.3) |  |
| **Ethnicity(%)** |  |  |  |  |  |  |  | 0.038 |
| **White** | 2592.5 (75.7) | 6149.3 (77.9) | 654.1 (77.2) | 1326.2 (77.8) | 95.3 (66.7) | 3484.6 (77.4) | 164.5 (75.9) |  |
| **Black** | 397.6 (11.6) | 753.5 ( 9.5) | 84.5 (10.0) | 159.1 ( 9.3) | 25.9 (18.1) | 440.8 ( 9.8) | 26.2 (12.1) |  |
| **Others** | 435.1 (12.7) | 993.2 (12.6) | 108.2 (12.8) | 220.4 (12.9) | 21.6 (15.1) | 574.5 (12.8) | 26.1 (12.0) |  |
| **Weight,kg** | 83.06 (24.52) | 82.76 (23.38) | 81.52 (21.76) | 82.33 (24.30) | 78.71 (21.27) | 82.75 (23.53) | 82.18 (23.35) | 0.248 |
| **First_care_unit(%)** |  |  |  |  |  |  |  | <0.001 |
| **MICU/SICU** | 1974.4 (57.6) | 4338.0 (54.9) | 480.6 (56.8) | 1007.8 (59.1) | 85.3 (59.7) | 2502.6 (55.6) | 134.6 (62.1) |  |
| **CCU** | 966.9 (28.2) | 2596.1 (32.9) | 242.7 (28.7) | 448.0 (26.3) | 34.2 (23.9) | 1458.5 (32.4) | 53.7 (24.8) |  |
| **Others** | 483.8 (14.1) | 961.9 (12.2) | 123.6 (14.6) | 249.9 (14.7) | 23.3 (16.3) | 538.8 (12.0) | 28.5 (13.2) |  |
| **SOFA** | 3.63 (2.07) | 3.73 (2.08) | 3.75 (2.06) | 3.71 (2.09) | 3.94 (2.01) | 3.75 (2.07) | 3.72 (2.07) | 0.207 |
| **Charlson_comorbidity_index** | 6.04 (2.83) | 5.87 (2.87) | 5.73 (2.78) | 6.03 (2.92) | 6.27 (3.13) | 5.85 (2.90) | 6.38 (3.15) | 0.002 |
| **Ventilation(%)** |  |  |  |  |  |  |  | 0.001 |
| **No** | 1896.8 (55.4) | 4144.1 (52.5) | 456.2 (53.9) | 946.6 (55.5) | 80.4 (56.3) | 2335.2 (51.9) | 141.9 (65.4) |  |
| **Yes** | 1528.4 (44.6) | 3751.9 (47.5) | 390.7 (46.1) | 759.1 (44.5) | 62.4 (43.7) | 2164.7 (48.1) | 74.9 (34.6) |  |
| **Vasopressor(%)** |  |  |  |  |  |  |  | <0.001 |
| **No** | 2485.5 (72.6) | 5593.0 (70.8) | 578.7 (68.3) | 1145.5 (67.2) | 94.7 (66.3) | 3083.1 (68.5) | 137.9 (63.6) |  |
| **Yes** | 939.7 (27.4) | 2303.0 (29.2) | 268.2 (31.7) | 560.3 (32.8) | 48.1 (33.7) | 1416.8 (31.5) | 78.9 (36.4) |  |
| **CRRT(%)** |  |  |  |  |  |  |  | 0.146 |
| **No** | 3346.2 (97.7) | 7739.1 (98.0) | 832.9 (98.4) | 1656.2 (97.1) | 138.0 (96.6) | 4403.1 (97.8) | 211.6 (97.6) |  |
| **Yes** | 79.0 ( 2.3) | 156.8 ( 2.0) | 13.9 ( 1.6) | 49.5 ( 2.9) | 4.9 ( 3.4) | 96.8 ( 2.2) | 5.2 ( 2.4) |  |
| **AG(mEq/L)** | 14.14 (4.01) | 14.06 (4.18) | 14.04 (4.20) | 14.24 (4.33) | 14.22 (3.77) | 14.15 (4.29) | 14.71 (4.04) | 0.31 |
| **Sodium(mEq/L)** | 138.64 (5.24) | 138.59 (5.21) | 138.47 (5.12) | 138.39 (5.28) | 138.49 (5.09) | 138.43 (5.31) | 137.85 (5.74) | 0.301 |
| **Potassium(mEq/L)** | 4.20 (0.70) | 4.22 (0.72) | 4.19 (0.66) | 4.23 (0.70) | 4.20 (0.64) | 4.22 (0.71) | 4.22 (0.75) | 0.718 |
| **Chloride(mEq/L)** | 104.97 (6.52) | 105.22 (6.48) | 105.36 (6.12) | 105.01 (6.62) | 105.22 (5.95) | 105.06 (6.53) | 104.45 (7.10) | 0.333 |
| **Bicarbonate(mEq/L)** | 23.28 (4.79) | 23.13 (4.90) | 22.88 (4.80) | 22.96 (4.91) | 22.70 (4.05) | 23.07 (4.85) | 22.56 (4.93) | 0.08 |
| **Phosphate(mEq/L)** | 3.67 (1.38) | 3.64 (1.40) | 3.62 (1.35) | 3.65 (1.42) | 3.64 (1.38) | 3.65 (1.43) | 3.75 (1.43) | 0.844 |
| **Glucose(mg/dL)** | 145.08 (68.70) | 142.20 (71.15) | 140.10 (63.81) | 139.08 (61.48) | 141.69 (56.76) | 141.26 (68.43) | 156.75 (213.57) | 0.021 |
| **Creatinine(g/dL)** | 1.59 (1.62) | 1.54 (1.54) | 1.46 (1.42) | 1.51 (1.46) | 1.57 (1.38) | 1.52 (1.50) | 1.57 (1.36) | 0.307 |
| **BUN(mg/dL)** | 29.38 (23.57) | 28.42 (22.82) | 27.43 (20.07) | 28.70 (22.03) | 30.90 (23.35) | 28.06 (22.27) | 30.00 (20.94) | 0.082 |
| **RBC(m/uL)** | 3.47 (0.66) | 3.43 (0.66) | 3.43 (0.65) | 3.42 (0.67) | 3.37 (0.62) | 3.42 (0.66) | 3.34 (0.65) | 0.017 |
| **WBC(k/uL)** | 12.73 (9.88) | 12.87 (8.23) | 13.10 (8.79) | 12.94 (7.67) | 12.84 (7.96) | 13.01 (8.83) | 13.52 (7.78) | 0.716 |
| **Platelet(k/uL)** | 199.69 (107.99) | 197.72 (114.94) | 197.47 (110.02) | 199.31 (112.31) | 197.03 (120.03) | 198.39 (113.72) | 205.17 (117.44) | 0.972 |
| **RDW(%)** | 15.32 (2.24) | 15.37 (2.26) | 15.40 (2.18) | 15.50 (2.33) | 15.80 (2.29) | 15.43 (2.30) | 15.72 (2.23) | 0.016 |
| **Hemoglobin(g/dL)** | 10.37 (1.96) | 10.26 (1.92) | 10.24 (1.88) | 10.27 (2.00) | 10.01 (1.74) | 10.24 (1.90) | 10.08 (1.95) | 0.025 |
| **pCO2(mmHg)** | 41.89 (12.45) | 41.64 (11.47) | 41.65 (11.01) | 41.96 (12.21) | 40.74 (9.33) | 41.78 (11.45) | 41.48 (11.56) | 0.775 |
| **pO2(mmHg)** | 130.77 (86.00) | 130.33 (85.49) | 131.25 (81.62) | 126.88 (84.83) | 133.69 (87.31) | 127.47 (83.51) | 128.69 (96.72) | 0.404 |
| **pH** | 7.37 (0.09) | 7.37 (0.09) | 7.37 (0.09) | 7.36 (0.09) | 7.36 (0.09) | 7.37 (0.09) | 7.37 (0.10) | 0.046 |
| **Shock_index1(min-1.mmHg-1)** | 0.63 (0.13) | 0.85 (0.15) | 1.35 (0.20) | 1.20 (0.19) | 1.97 (0.50) | 0.97 (0.15) | 1.46 (0.28) | <0.001 |
| **Shock_index2(min-1.mmHg-1)** | 0.61 (0.10) | 0.81 (0.13) | 1.13 (0.21) | 1.21 (0.19) | 1.46 (0.47) | 0.98 (0.14) | 1.53 (0.32) | <0.001 |
| **Shock_index3(min-1.mmHg-1)** | 0.60 (0.10) | 0.79 (0.11) | 0.96 (0.17) | 1.22 (0.19) | 1.17 (0.23) | 0.99 (0.14) | 1.53 (0.29) | <0.001 |
| **Shock_index4(min-1.mmHg-1)** | 0.60 (0.10) | 0.78 (0.11) | 0.88 (0.14) | 1.22 (0.18) | 1.05 (0.17) | 1.00 (0.14) | 1.56 (0.30) | <0.001 |
| **Shock_index5(min-1.mmHg-1)** | 0.60 (0.10) | 0.78 (0.11) | 0.84 (0.13) | 1.22 (0.21) | 1.02 (0.19) | 0.99 (0.14) | 1.55 (0.35) | <0.001 |
| **Shock_index6(min-1.mmHg-1)** | 0.60 (0.11) | 0.77 (0.12) | 0.83 (0.14) | 1.18 (0.20) | 1.03 (0.19) | 0.99 (0.16) | 1.45 (0.31) | <0.001 |
| **Ventilation-free days in 28 days,day** | 25.46 (3.62) | 25.56 (3.39) | 25.33 (3.47) | 24.84 (3.88) | 24.00 (4.85) | 25.16 (3.89) | 24.68 (4.62) | <0.001 |
| **Vasopressor-free days in 28 days,day** | 27.40 (1.81) | 27.31 (1.97) | 27.31 (1.64) | 26.90 (2.50) | 26.96 (1.93) | 27.10 (2.29) | 26.62 (3.27) | <0.001 |
| **Death in 28 days(%)** |  |  |  |  |  |  |  | <0.001 |
| **No** | 3007.4 (87.8) | 6986.7 (88.5) | 731.6 (86.4) | 1324.3 (77.6) | 104.1 (72.9) | 3799.7 (84.4) | 154.9 (71.4) |  |
| **Yes** | 417.8 (12.2) | 909.2 (11.5) | 115.3 (13.6) | 381.4 (22.4) | 38.8 (27.1) | 700.2 (15.6) | 61.9 (28.6) |  |
| **Death in hospital(%)** |  |  |  |  |  |  |  | <0.001 |
| **No** | 2981.6 (87.0) | 6914.5 (87.6) | 719.5 (85.0) | 1303.0 (76.4) | 102.6 (71.9) | 3744.0 (83.2) | 151.5 (69.9) |  |
| **Yes** | 443.6 (13.0) | 981.5 (12.4) | 127.4 (15.0) | 402.8 (23.6) | 40.2 (28.1) | 755.9 (16.8) | 65.3 (30.1) |  |
| **Death in ICU(%)** |  |  |  |  |  |  |  | <0.001 |
| **No** | 3121.5 (91.1) | 7237.2 (91.7) | 766.5 (90.5) | 1424.8 (83.5) | 113.6 (79.5) | 4010.3 (89.1) | 173.9 (80.2) |  |
| **Yes** | 303.7 ( 8.9) | 658.7 ( 8.3) | 80.4 ( 9.5) | 281.0 (16.5) | 29.2 (20.5) | 489.6 (10.9) | 42.9 (19.8) |  |
| **Length of stay in hospital,day** | 11.73 (13.70) | 11.16 (11.79) | 14.04 (49.71) | 13.05 (13.44) | 13.18 (11.81) | 11.82 (12.89) | 13.48 (12.85) | <0.001 |
| **Length of stay in ICU,day** | 5.85 (6.82) | 5.20 (6.43) | 5.60 (10.03) | 6.16 (6.35) | 7.05 (7.22) | 5.65 (6.88) | 6.78 (7.84) | <0.001 |

Abbreviations: IPTW, inverse probability of treatment weighting; MICU, medical intensive care unit; SICU, surgical intensive care unit; CCU, coronary care unit; SOFA, Sequential Organ Failure Assessment; CRRT, continuous renal replacement therapy; AG, anion gap; BUN, blood urea nitrogen; RBC, red blood cells; WBC, white blood cells; RDW, red blood cell distribution width.

Table S5. Results of double robust analysis.

| **Class** | **Propensity score IPTW** | | **Doubly robust with unbalanced covariates** | | **Doubly robust with all covariates** | |
| --- | --- | --- | --- | --- | --- | --- |
|  | **HR(95%CI)** | **P-value** | **HR(96%CI)** | **P-value** | **HR(96%CI)** | **P-value** |
| **class1** | Reference |  | Reference |  | Reference |  |
| **class2** | 1.023(0.906-1.154) | 0.718 | 1.015(0.897-1.148) | 0.818 | 1.013(0.894-1.147) | 0.843 |
| **class3** | 1.103(0.897-1.356) | 0.352 | 1.115(0.903-1.377) | 0.312 | 1.112(0.899-1.376) | 0.326 |
| **class4** | 1.636(1.417-1.890) | <0.001 | 1.650(1.426-1.909) | <0.001 | 1.674(1.443-1.942) | <0.001 |
| **class5** | 2.023(1.361-3.006) | <0.001 | 2.035(1.385-2.990) | <0.001 | 2.057(1.410-3.002) | <0.001 |
| **class6** | 1.296(1.144-1.468) | <0.001 | 1.291(1.136-1.467) | <0.001 | 1.299(1.143-1.477) | <0.001 |
| **class7** | 2.132(1.494-3.041) | <0.001 | 2.076(1.454-2.963) | <0.001 | 2.220(1.547-3.186) | <0.001 |

Abbreviations: HR, hazard ratio; CI, confidence interval.

Table S6. Results of subgroup analysis.

| **Subgroups** | **No.of death/No.of patients** | **class1** | **class2** | **class3** | **class4** | **class5** | **class6** | **class7** | **P for interaction** |
| --- | --- | --- | --- | --- | --- | --- | --- | --- | --- |
| **Age** |  |  |  |  |  |  |  |  | 0.423 |
| **<65** | 1053/8539 | Reference | 0.988(0.800-1.218) | 1.391(1.023-1.891) | 1.648(1.312-2.070) | 1.812(1.199-2.739) | 1.237(0.999-1.532) | 2.796(2.056-3.800) |  |
| **≥65** | 2005/11330 | Reference | 1.005(0.879-1.148) | 1.041(0.831-1.303) | 1.758(1.500-2.061) | 1.902(1.375-2.632) | 1.311(1.138-1.511) | 2.247(1.690-2.987) |  |
| **Gender** |  |  |  |  |  |  |  |  | 0.168 |
| **Male** | 1698/11615 | Reference | 1.058(0.911-1.228) | 1.387(1.094-1.759) | 1.699(1.427-2.024) | 2.093(1.508-2.905) | 1.293(1.105-1.513) | 2.445(1.861-3.210) |  |
| **Female** | 1360/8254 | Reference | 0.944(0.795-1.121) | 0.953(0.721-1.258) | 1.804(1.484-2.193) | 1.715(1.141-2.578) | 1.335(1.117-1.595) | 2.994(2.198-4.076) |  |
| **Ethnicity** |  |  |  |  |  |  |  |  | 0.946 |
| **White** | 2373/15320 | Reference | 1.006(0.884-1.143) | 1.124(0.913-1.384) | 1.714(1.479-1.986) | 1.939(1.449-2.595) | 1.272(1.111-1.456) | 2.590(2.056-3.262) |  |
| **Black** | 310/2013 | Reference | 1.128(0.793-1.605) | 1.398(0.806-2.424) | 1.654(1.076-2.542) | 2.573(1.224-5.411) | 1.657(1.153-2.381) | 3.635(1.913-6.908) |  |
| **Other** | 375/2536 | Reference | 0.932(0.678-1.281) | 1.191(0.724-1.960) | 1.854(1.270-2.706) | 1.614(0.742-3.508) | 1.292(0.930-1.795) | 1.914(1.040-3.524) |  |
| **Charlson_comorbidity_index** |  |  |  |  |  |  |  |  | 0.024 |
| **<6** | 890/9300 | Reference | 0.812(0.658-1.002) | 0.901(0.656-1.237) | 1.306(1.023-1.669) | 1.470(0.948-2.281) | 0.874(0.698-1.095) | 1.856(1.315-2.618) |  |
| **≥6** | 2168/10569 | Reference | 1.075(0.941-1.228) | 1.185(0.951-1.478) | 1.904(1.633-2.221) | 2.043(1.492-2.797) | 1.485(1.293-1.706) | 2.953(2.285-3.816) |  |
| **SOFA** |  |  |  |  |  |  |  |  | 0.199 |
| **<3** | 791/7050 | Reference | 1.159(0.951-1.413) | 1.224(0.848-1.769) | 1.756(1.365-2.259) | 2.021(1.079-3.785) | 1.205(0.967-1.502) | 1.947(1.164-3.257) |  |
| **≥3** | 2267/12819 | Reference | 0.950(0.829-1.089) | 1.157(0.939-1.424) | 1.721(1.476-2.006) | 1.933(1.460-2.561) | 1.334(1.159-1.535) | 2.673(2.126-3.360) |  |
| **Vasopressor** |  |  |  |  |  |  |  |  | 0.526 |
| **No** | 1626/13853 | Reference | 1.099(0.961-1.258) | 1.147(0.862-1.526) | 1.764(1.478-2.107) | 1.738(1.050-2.877) | 1.382(1.194-1.599) | 2.349(1.507-3.664) |  |
| **Yes** | 1432/6016 | Reference | 0.839(0.682-1.031) | 1.031(0.797-1.334) | 1.573(1.275-1.941) | 1.735(1.258-2.394) | 1.143(0.931-1.404) | 2.452(1.880-3.197) |  |

Abbreviations: SOFA, Sequential Organ Failure Assessment; CRRT, continuous renal replacement therapy.

Table S7. Secondary outcome analysis.

| **Secondary outcomes** | **In-ICU death** | | **28-day death** | | **Ventilation-free days in 28 days** | | **Vasopressor-free days in 28 days** | |
| --- | --- | --- | --- | --- | --- | --- | --- | --- |
|  | **HR(95%CI)** | **P-value** | **HR(95%CI)** | **P-value** | **β(95%CI)** | **P-value** | **β(95%CI)** | **P-value** |
| **class1** | Reference |  |  |  |  |  |  |  |
| **class2** | 1.052(0.916-1.210) | 0.473 | 0.993(0.884-1.115) | 0.898 | 0.117(-0.022-0.256) | 0.099 | -0.156(-0.234--0.078) | <0.001 |
| **class3** | 1.182(0.951-1.470) | 0.133 | 1.153(0.957-1.389) | 0.135 | -0.128(-0.383-0.127) | 0.325 | -0.461(-0.603--0.319) | <0.001 |
| **class4** | 1.714(1.464-2.007) | <0.001 | 1.732(1.516-1.980) | <0.001 | -0.529(-0.729--0.329) | <0.001 | -0.844(-0.955--0.734) | <0.001 |
| **class5** | 1.945(1.462-2.589) | <0.001 | 1.934(1.494-2.503) | <0.001 | -0.972(-1.457--0.487) | <0.001 | -0.967(-1.239--0.696) | <0.001 |
| **class6** | 1.236(1.068-1.431) | 0.005 | 1.287(1.140-1.453) | <0.001 | -0.254(-0.409--0.099) | 0.001 | -0.447(-0.534--0.360) | <0.001 |
| **class7** | 2.361(1.868-2.983) | <0.001 | 2.655(2.152-3.277) | <0.001 | -1.190(-1.603--0.778) | <0.001 | -1.602(-1.831--1.372) | <0.001 |

All covariables were adjusted in the models of Death in ICU and Death in 28 days; All covariables except ventilator were adjusted in the Model of Ventilation-free days in 28 days; All covariables except ventilator were adjusted in the Model of Vasopressor-free days in 28 days.
